# Giant peripheral osteoma of the mandible simulating a parotid gland tumor^☆^

**DOI:** 10.1016/j.bjorl.2016.03.004

**Published:** 2016-04-22

**Authors:** Javier Ata-Ali, Fadi Ata-Ali

**Affiliations:** a Hospital Universitari Arnau de Vilanova, Servicio Público de Salud Dental, Valencia, Spain; b European University of Valencia, Departamento de Odontología, Valencia, Spain; c Private Practice, Valencia, Spain

## Introduction

Osteoma is a benign osteogenic lesion characterized by the proliferation of compact or cancellous bone. It can be central, peripheral or extraskeletal.^1^ Peripheral osteomas are defined by centrifugal growth from the periosteum, while central osteomas arise centripetally from the endosteum.^1^, ^2^

The pathogenesis of peripheral osteoma is unclear.^1^ However, a common path underlying the developmental process of osteomas has been recognized: it has been supposed that a combination of a trauma and muscle activity can initiate an osteogenic reaction.^3^ In the course of their slow but steady increase in size, osteomas of the maxillofacial bones remain asymptomatic until they attain sufficient sizes as to cause disfigurement and/or direct interference with the normal function of their anatomic location.^4^

We report the case of a peripheral osteoma simulating a parotid gland tumor due to its location and size, and the presence of infrequent clinical manifestations.

## Case report

A 67-year-old woman was referred to our hospital due to a tumor of benign appearance located in the left parotid region, and which according to the patient had been present for one year. The lesion had neither increased nor decreased in size since she first became aware of its presence. The patient experienced no dysphagia or trismus, and had no history of cervicofacial trauma.

The physical examination revealed a firm and well-circumscribed mass in the region of the left parotid gland, covered by normal skin without ulceration or color changes. Facial nerve function proved normal, and the patient only experienced a sensation of increased volume in the zone, without pain.

**Figure 1:**
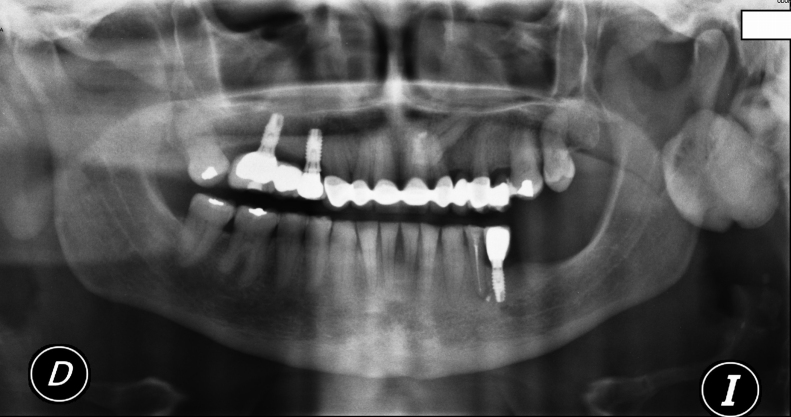
Panoramic X-ray view showing a radiopaque lesion located in the middle of the left mandibular ascending ramus.

**Figure 2:**
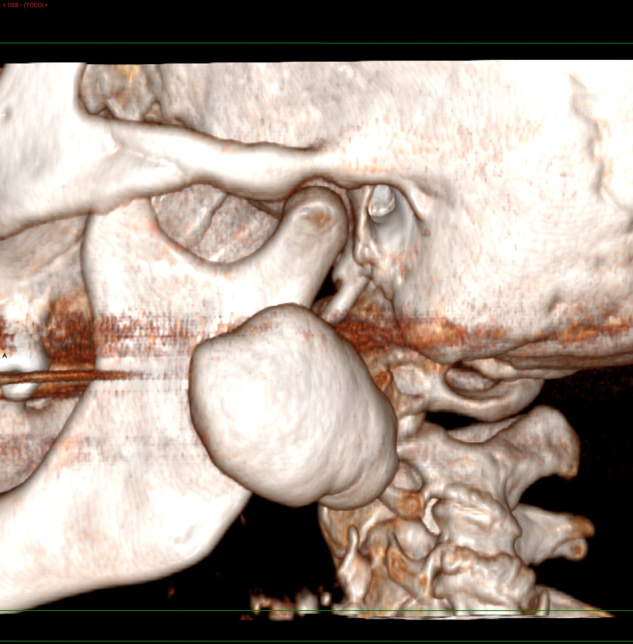
Three-dimensional image showing a perfectly delimited lesion of exophytic benign appearance in the external cortical layer of the mandibular ascending ramus.

**Figure 3:**
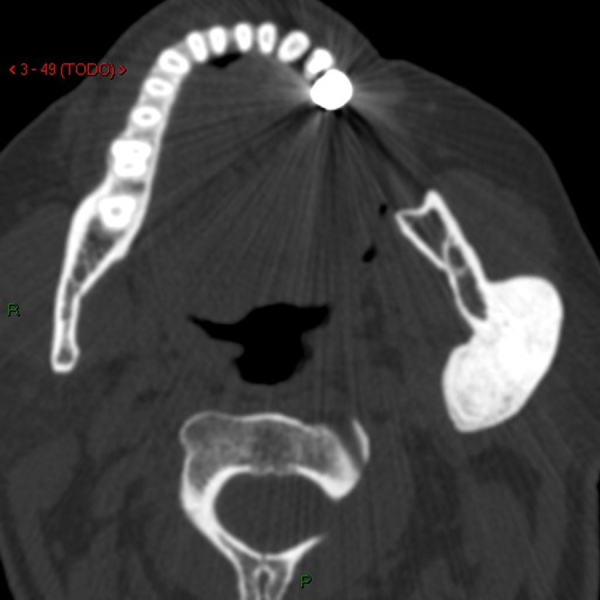
Axial computed tomography scan showing the lesion to extend toward the internal and external surfaces of the mandibular ascending ramus, causing no bone reabsorption or destruction.

A fine needle aspiration biopsy (FNAB) was requested but was unable to extract material for study. Imaging techniques were therefore decided, and the panoramic X-ray study revealed a radiopaque lesion located in the middle of the left mandibular ascending ramus (Fig. 1). Three-dimensional imaging showed the lesion to be perfectly delimited within the external cortical bone layer (Fig. 2). The axial computed tomography scan showed the lesion to extend toward the internal and external surfaces of the mandibular ascending ramus, causing no bone reabsorption or destruction (Fig. 3).

The patient declined surgery. Conservative management was therefore decided, with periodic clinical and radiographic controls.

## Discussion

The first diagnostic impression was a parotid tumor, in view of the location of the peripheral osteoma in the external zone of the mandibular ascending ramus, causing protrusion of the parotid gland and adjacent soft tissues.

Peripheral osteoma is a rare neoplasia in the maxillofacial region and it more frequently involves the mandible, where the condyle is the preferred site.^5^ Solitary osteoma can be classified as peripheral (parosteal, periosteal or exophytic) when arising from the periosteum (as in our patient), central (endosteal) when arising from the endosteum, or extraskeletal (so-called osseous choristoma) when arising in soft tissue.^6^ Osteomas must be well identified and differentiated from other solid diseases.^3^ Radiographically, osteoma is characterized by an oval, radiopaque, well-circumscribed mass attached by a broad base or pedicle to the bone cortex.^1^, ^2^

The treatment of incidentally found asymptomatic osteomas is controversial.^7^ The slow growth of osteomas allows a conservative attitude toward asymptomatic lesions and surgical treatment is not recommended in this asymptomatic cases.^8^ The decision to surgically remove the lesion should be based on adequate evaluation of the risks of the operation, including possible damage to important anatomical structures.

Burke et al.^9^ concluded that ultrasound is an inexpensive, precise and accessible imaging option involving no ionizing radiation and capable of distinguishing between benign and malignant lesions in up to 90% of the cases, as well as between sialoliths and cystic lesions.

In the event of an apparent parotid gland tumor, a panoramic X-ray study or ultrasound evaluation might be indicated before deciding FNAB, since such imaging studies are simple, noninvasive and inexpensive, and can identify possible bone disease – thereby avoiding unnecessary diagnostic procedures such as FNAB.

## Conclusions

Peripheral mandibular osteoma can be included in the differential diagnosis of parotid gland tumors. In such situations, an initial panoramic X-ray or ultrasound evaluation is indicated, with a view to avoiding possibly unnecessary procedures such as FNAB.

## Conflicts of interest

The authors declare no conflicts of interest.
